# Study on Thermodynamic Characteristics and Heat Transfer Method of Uncontrolled Fire in Coal Mine Gangue Mountain Spontaneous Combustion Based on System Dynamics

**DOI:** 10.1155/2022/5953322

**Published:** 2022-08-26

**Authors:** Guangxing Bai

**Affiliations:** College of Safety Science and Engineering, Xi'an University of Science and Technology, Shaanxi, Xi'an 710054, China

## Abstract

Coal fire disaster caused by the spontaneous combustion of coal has always been one of the serious problems that threaten the safety of coal mining. In our study, we first solved the dynamic parameters and mechanical functions of coal gangue using the Achar differential method and Coats-Redfern integral method. Then, based on the flow and heat transfer mechanism of hot rod vapor-liquid two-phase flow, combined with coal spontaneous ignition conditions, influencing factors, coal pile spontaneous combustion temperature field structure distribution, etc., the heat transfer process of the hot rod in the coal pile (coal gangue mountain) was analyzed. Results show the average activation energies of the second stage of different types of coal mine gangue. Upon comparing the characteristic parameters of coal gangue in different regions and ages, it is found that 8# coal gangue has better combustion and burn-out characteristics, and its comprehensive combustion characteristics are second only to those of the coal gangue in Datong and higher than those in Panzhihua, Pingdingshan, and Hancheng. The higher the content of volatile matter and fixed carbon in coal gangue, the lower the ash content, and the better the comprehensive combustion performance of the coal gangue. Under the condition of sufficient oxygen supply combustion, the larger the fuel ratio, the better the burnout performance of the coal gangue. The test of the influence of the hot rod on the temperature field distribution inside the coal pile shows that the maximum cooling rate of a single hot rod to the coal pile during the test period is 33.4°C, and the maximum cooling rate reaches 39.6%. The calculated heat dissipation of the 80 h hot rod is 1.0865, 2.1680, and 3.3649 MJ, respectively.

## 1. Introduction

Coal fire disasters caused by the spontaneous combustion of coal have always been one of the serious problems threatening the safe mining of coal mines. Coal fire disasters spread all over the world, such as in China, Australia, Poland, Portugal, the United States, India, etc [1]. Coal fires caused by the spontaneous combustion of coal burn a lot of resources every year and emit greenhouse gases and toxic and harmful gases, producing CO, CO_2_, CH_4_, SO_2_, NO_*X*_, etc [2, 3]. The CO_2_ produced by coal fires in China accounts for about 0.1%–0.22% of global carbon emissions from fossil fuels. The emissions from these coal fires pollute the atmosphere, destroy the ozone layer, cause the greenhouse effect and global warming, and seriously threaten ecological security. Therefore, controlling the coal fires caused by the spontaneous combustion of coal has become a huge issue facing the world today [4]. The spontaneous combustion of coal gangue is a relatively special combustion system, which has the characteristics of large heat storage and easy reignition. Coal and coal gangue mountains stacked in the open often spontaneously combust, resulting in the wastage of resources, environmental pollution, and serious accidents. Aiming at the key scientific problems in the prevention and control of spontaneous combustion of coal gangue mountain, based on the research idea of “heat transfer and cooling,” the prevention and control methods are carried out focusing on the kinetic characteristics of uncontrolled spontaneous combustion and heat transfer process of coal gangue mountain. The scientific management of fire has important theoretical guiding significance.

The research on the spontaneous combustion mechanism of coal began in the 17^th^ century, however, the research on the spontaneous combustion mechanism of the coal gangue only started in the late 19^th^ century [5]. As humans find that the hazard of the spontaneous combustion of coal gangue is becoming more serious and the world has begun to pay attention to the concept of environmental protection, scientists in various professional fields from all over the world have carried out in-depth research on the spontaneous combustion mechanism of the coal gangue since the 21^st^ century [6]. The spontaneous combustion of coal gangue is a complex physical and chemical reaction process, which has been an important subject of human exploration and research for centuries. In the 17^th^ century, people believed that coal gangue was formed because of the reaction of pyrite (FeS2) in the air to generate FeSO_4_, Fe_2_(SO_4_)_3_, Fe(OH)_3_, SO_2_, CO_2_, CO, H_2_S, and other products. At the same time, heat gradually warmed the coal. Eventually, it led to the self-heating and spontaneous combustion of coal gangue. Hence, the theory of spontaneous combustion caused by pyrite is proposed [7]. In 1870, Rachtan discovered through experiments that coal had oxygen-absorbing properties. Jones (Jones E·R) further confirmed the oxygen absorption of coal in 1945, pointing out that bituminous coal can absorb 0.4 ml/g oxygen when exposed to air at room temperature. In the 1960s, the Fushun Coal Research Institute of China proposed a chromatographic oxygen absorption identification method for the spontaneous combustion tendency of coal. In the 1990s, Australian Chinese scholars Humphreys and Ren et al. proposed to use the average self-heating rate index R70 (self-heating rate index) of the adiabatic heating process of coal between 40°C and 70°C to characterize the spontaneous combustion tendency of coal. After that, the R70 indicator to measure the spontaneous combustion tendency of coal has been widely studied and applied in many countries, such as Australia, New Zealand, and China [8].

In recent years, with the development of science and technology and the continuous enrichment of technical means, many advanced experimental techniques and methods, such as adiabatic heating, temperature programming, the spontaneous combustion of large coal, thermal analysis, in situ spectroscopy and thermal analysis, and quantum chemistry theory have been successively adopted. It is used to study the mechanism of coal spontaneous combustion and reveal the micromechanism and macrodynamic characteristics of the coal spontaneous combustion process [9]. For example, Ren et al. studied the relationship between the size of coal spontaneous combustion and the number of active groups [10], and Beamish et al., based on the quantum chemical theory method, simulated and calculated the active order of coal active groups to reveal the nature of coal spontaneous combustion [11].

The current research contents on the coal gangue mountain control at home and abroad mainly include the following points: research on the stability of coal gangue mountain slopes, research on the prevention and control technology of coal gangue mountain spontaneous combustion, research on mountain antipollution treatment and transformation technology, research on restoration and treatment of coal gangue mountain greening environment, etc. [12]. The fire-fighting methods of the gangue mountain mainly include excavating the fire source method, hydraulic fire-fighting method, wrapping method, covering fire-fighting method, and grouting fire-fighting method [13]. The two conditions of sulfur combustibles, ventilation, and oxygen supply are as follows: make full use of flame retardant and water in filling the slurry to reduce the temperature of the coal gangue in the deep area, wrap sulfur-containing combustibles with mud and lime slurry, fill the gaps, and isolate oxygen to reduce the temperature of coal gangue in deep areas. To achieve the purpose of blocking combustion, cooling, and extinguishing, the coal gangue no longer has the combustion conditions [14].

The waste heat resource is the residual heat after the combustion of primary energy and combustibles and is regarded as the fifth largest energy source after coal, oil, natural gas, and water power. China's energy utilization rate is about 10% lower than that of developed countries, and at least more than half of the industrial energy consumption is directly discarded in the form of waste heat [15]. Waste heat resources can be divided into three types according to temperature grade: low-temperature waste heat (<300°C), medium-temperature waste heat (300−600°C), and high-temperature waste heat (>600°C). The residual heat energy in spontaneous combustion coal gangue hills and coalfield fire areas is the heat stored after coal combustion and has a wide temperature distribution range, a wide fire area, and a large amount of heat storage, which has potential utilization. At present, the methods for cooling the spontaneous coal gangue mountain fire areas include water injection, blasting, dry ice, liquid nitrogen injection, liquid CO_2_ injection, grouting, glue injection, etc., which belong to the phase change absorption heat of the water or inert gas to reduce the high-temperature fire zone temperature [16]. Liquid nitrogen cooling and fire extinguishing technology have been used earlier in the field of mine fire-fighting. With its excellent low-temperature cooling and safety characteristics, colorless, odorless, noncorrosive, and low temperature liquid nitrogen has become an important fire-fighting technical means and has now formed a mature technical system, equipment, and process [17].

In 1942, Gaugler proposed the heat pipe (HP) by envisioning the manufacture of refrigerators. The heat pipe is a high-efficiency heat transfer element with gas-liquid two-phase flow circulation [18]. It was invented in the 1960s to solve the special requirements for heat transfer in spaceflight. Because of its huge role in energy conservation, its theoretical research and engineering applications have made great achievements in temperature uniformity, temperature control, waste heat recovery, new energy development, etc., and they have been widely used in chemical, energy, power, and mining fields [19]. In terms of the engineering application research of hot rods, the Institute of Cold Regions of the Chinese Academy of Sciences, Lanzhou University, and other units have successively carried out research on the thermal stability of hot rods to protect frozen soil subgrades in the Qingshui River section of the Qinghai-Tibet Railway and found that the effective heat transfer radius of the hot rods is 1.5 m, the maximum heat transfer influence range is 2.16 m, the numerical simulation shows that the installation distance between the heat pipes can be designed to be 3.3–3.8 m, and the upper limit of the frozen soil of the subgrade can be raised by 0.8–1.2 m [20].

The research on the heat transfer and cooling of hot rods in the coal gangue mountains or coal piles began in the 1990s. In 1991, P. Muthukumar and M. Groll experimentally tested the cooling effect of gravity hot rods on the coal pile in the Hepingmen coal yard of Nanjing Fuel Company. Compared with the dense-hole cooling method, the hot rod is more effective in preventing coal spontaneous combustion [21]. A. Adamus established a heat transfer calculation model for hot rods from the perspective of engineering design and analyzed the technical and economic feasibility of using hot rods for heat transfer in deep coal gangue mountains. The first stage is inserted into the area where the temperature does not exceed 250°C. The second stage is inserted into the self-ignition center area by staged cooling measures [22]. In 2014, M. Mochizuki et al. discussed the advantages of using hot rods in the prevention and control of coal gangue mountains, analyzed the technical difficulties of using hot rods in the spontaneous combustion of coal gangue mountains to remove heat and cool down, and studied the heat transfer and cooling of coal spontaneous combustion prevention and fire-extinguishing heat rods. The performance test method shows that the cooling ability of the hot rod to the coal pile decreases with the increase of the distance, the cumulative heat transfer and cooling ability of the hot rod to the coal pile increase with time, and the radius of the distance to the hot rod is 20 mm. The cooling effect is the most significant, with a maximum temperature reduction of 13.3°C, a decrease of up to 29% [23].

A large number of pieces of research have been carried out on the spontaneous combustion characteristics, process, and prevention and control methods of coal gangue mountains at home and abroad, however, the research on the spontaneous combustion mechanism of the coal gangue and its heat transfer prevention and control methods mainly have the following shortcomings: (1) influenced by the thermal storage environment, oxygen supply conditions, and self-oxidation characteristics, the kinetic process of coal gangue oxidation and spontaneous combustion is complex, and there are many influencing factors, which are typically noncontrollable. It is difficult for existing research to accurately determine the high temperature area in the coal gangue mountain and its development trend. (2) The unsteady characteristics of heat generation and accumulation during the spontaneous combustion of coal gangue mountains cannot be accurately grasped, and it is difficult to reveal the kinetic mechanism of heat transfer during the spontaneous combustion of coal gangue mountains, and the kinetic mechanism of nonheat-controlled combustion of coal gangue is unclear.

To this end, this study plans to use a combination of theoretical analysis, experimental testing, and numerical simulation to build a coal gangue multifield-coupled uncontrolled combustion kinetic model based on the characteristics of the thermal migration behavior of coal gangue mountain spontaneous combustion. The research provides theoretical guidance for grasping the spontaneous combustion mechanism of coal gangue hills and is of great significance for preventing and controlling spontaneous combustion and the reignition of coal gangue hills.

At present, a large number of studies have been carried out on the spontaneous combustion characteristics, processes, and prevention and control methods of coal gangue mountains at home and abroad, however, there are several deficiencies in the research on the spontaneous combustion mechanism of coal gangue and its heat transfer prevention and control methods, which are as follows: (1) there are few studies on the spontaneous combustion characteristic parameters, such as gas index, critical temperature, and oxygen consumption rate, in the spontaneous combustion process of coal gangue. (2) Existing research is difficult to accurately determine the high temperature area and its development trend inside the coal gangue mountain. (3) The unsteady characteristics of heat generation and accumulation during the spontaneous combustion of coal gangue mountains cannot be accurately grasped, and it is difficult to reveal the kinetic mechanism of heat transfer during the spontaneous combustion of coal gangue mountains. The kinetic mechanism of the non-heat-controlled combustion of the coal gangue is unclear. (4) There are few pieces of research on key parameters and methods, such as heat transfer technical characteristics, heat transfer radius, and heat transfer effect evaluation of hot rods, implanted in coal gangue mountains, and the basic parameters for the directional heat transfer control of hot rods in the coal gangue mountain fire areas have not been determined.

Therefore, in our study, we first introduced the research methods and thermodynamic theory including similarity theory, fluid mechanics, thermodynamics, heat transfer, numerical heat transfer, and other theories in Section 2. Then, we conducted the coal gangue reaction kinetics solution, the analysis of the kinetic parameters of coal gangue oxidative combustion, the analysis of comprehensive combustion characteristics of spontaneous combustion coal gangue, and a study on the heat transfer method of the hot rod in the gangue mountain in the results and discussion section.

## 2. Research Methods and Thermodynamic Theory

### 2.1. Research Methods

Using the similarity theory, fluid mechanics, thermodynamics, heat transfer, numerical heat transfer, and other theories, by analyzing the law of the natural ignition of the coal gangue mountains, and according to the flow and heat transfer law of vapor-liquid two-phase flow inside the hot rod, the heat transfer theory and the methods of the hot rod are organically combined with the spontaneous combustion mechanism and temperature field distribution of the gangue mountain to master the temperature field distribution law, influencing factors, and the ignition law of the gangue mountain oxidation and spontaneous combustion process, determine the conditions and influencing factors of gangue spontaneous combustion, and determine the dangerous area of the gangue mountain.

Using a variety of advanced experimental methods, such as industry analysis, chemical composition analysis, element analysis, and thermal physical parameter analysis, experimental research on coal gangue was carried out, and the correlation between the internal parameters of the coal gangue and the natural ignition characteristics was obtained. The trend of temperature change, combined with the measured parameters of the coal gangue, and the results of the experimental analysis are used to study the spontaneous ignition mechanism of the coal gangue.

### 2.2. Thermodynamic Theory

The process of the spontaneous combustion of the coal gangue starts from the accumulation of heat. The moisture and adsorbed free gas on the surface and pores of the gangue particles evaporate and desorb, and then, they oxidize and separate into a volatile matter. When the outside temperature is high enough and there is sufficient oxygen supply, the volatile matter burns, and the fixed carbon is cracked and ignited under high-temperature burning. The oxidative combustion of the coal gangue is controlled by chemical kinetics, in which the precipitation of volatiles controls the combustion process. In the process of the thermal analysis of coal, the relationship between physical parameters, such as coal mass and heat, and the reaction rate can be expressed in the following two forms:

integral form:
  (1) $$\begin{array}{c} M\left(\alpha \right)=kt \end{array}$$
differential form:
  (2) $$\begin{array}{c} \frac{d\alpha }{\text{d}t}=kf\left(\alpha \right), \end{array}$$
where *α* is the conversion rate of coal at time *t*, *k* is the reaction rate constant, *M*(*α*) is the integral form of the reaction mechanism function, and (*α*) is the differential form of the reaction mechanism function. The relationship between *f*(*α*) and *M*(*α*) can be expressed as follows:
  (3) $$\begin{array}{c} f\left(\alpha \right)=\frac{1}{M^{\prime }\left(\alpha \right)}=\frac{1}{d\left[M\left(\alpha \right)\right]/d\left(\alpha \right)}. \end{array}$$

The relationship between *k* and the reaction temperature *T* (absolute temperature) can be expressed according to the Arrhenius equation as follows:

(4) $$\begin{array}{c} k=A\times \;\exp\left(\frac{-E}{RT}\right), \end{array}$$

where *E* is the apparent activation energy of the reaction, kJ/mol. *A* is the apparent pre-exponential factor of the reaction, *R* is the universal gas constant, i.e., 8.314 J/(mol·K), and *T* is the absolute temperature.

Since the experimental sample is under the temperature-programmed experimental conditions of a certain heating rate, the relationship between the thermodynamic temperature and the time of the experimental sample is as follows:

(5) $$\begin{array}{c} T=T_{0}+at, \end{array}$$

where *T*_0_ is start temperature, K, and *a* is temperature rate, K·min^−1^. Therefore, the common kinetic equations for heterogeneous systems under nonisothermal conditions can be obtained.

The differential form is as follows:

(6) $$\begin{array}{c} \frac{d\alpha }{dT}=\frac{A}{a}f\left(\alpha \right)\exp\left(\frac{-E}{RT}\right). \end{array}$$

The integral form is as follows:

(7) $$\begin{array}{c} M\left(\alpha \right)=\int_{T_{0}}^{T}\frac{A}{a}\exp\left(-\frac{E}{RT}\right)dT\approx \int_{0}^{T}\frac{A}{a}\exp\left(-\frac{E}{RT}\right)dT=\frac{AE}{aR}H\left(u\right). \end{array}$$

In the calculation process of the nonisothermal kinetic equation used, the calculation of *H*(*u*) does not converge. Hence, it does not have an accurate analytical formula. In this study, the general kinetic analysis method of the heating rate under nonisothermal conditions is adopted, and the fitted correlation coefficients are consistent. The dynamic mode function and parameters of the coal gangue at different stages can be determined by the characteristics.

Achar integral method. Upon transforming equation (6), we can obtain the following another form:

(8) $$\begin{array}{c} \frac{a}{f\left(\alpha \right)}\frac{d\alpha }{dT}=A\;\exp\left(-\frac{E}{RT}\right). \end{array}$$

Upon taking the logarithm of both sides directly, we obtain the following:

(9) $$\begin{array}{c} \ln\frac{d\alpha }{f\left(\alpha \right)dT}=\ln\frac{A}{a}-\frac{E}{RT}. \end{array}$$

Equation (9) is called as the Achar–Brindley–Sharp–Wendworth equation.

(10) $$\begin{array}{c} \frac{d\alpha /\text{d}t}{f\left(\alpha \right)}=\ln\;A-\frac{E}{RT}. \end{array}$$

Based on (10), the relationship between d*α*/d*t*/*f*(*α*) and 1/*T* is linear. By substituting different solid reaction mechanism functions, a linear fitting analysis is performed to solve the corresponding reaction apparent activation energy *E*, pre-exponential factor *A*, and mechanism function *f* (*α*).

The Coats-Redfern integration method for activation energy is as follows:

For equation (1), a common kinetic equation in heterogeneous systems is as follows:

(11) $$\begin{array}{c} \frac{d\alpha }{dT}=\frac{A}{a}\exp\left(-E/RT\right)f\left(\alpha \right). \end{array}$$

According to the Coats-Redfern method,

(12) $$\begin{array}{c} \ln\left[\frac{M\left(\alpha \right)}{T^{2}}\right]=\ln\left(\frac{AR}{aE}\left(1-\frac{2RT}{E}\right)\right)-\frac{E}{RT}. \end{array}$$

where *α*=*m*_0_ − *m*/*m*_0_ − *m*_*∞*_ is reaction conversion, and *R* = 8.314 J·mol^−1^·K^−1^. Because of the general reaction temperature range and most *E* values, *E*/*RT* ≥ 1, 1 − 2*RT*/*E* ≈ 1, and (12) can be changed to the following:

(13) $$\begin{array}{c} \ln\left[\frac{M\left(\alpha \right)}{T^{2}}\right]=\ln\left(\frac{AR}{aE}\right)-\frac{E}{RT}. \end{array}$$

According to equation (13), integral form of the reaction mechanism function can be obtained.

Coal gangue is a complex mixture, and its spontaneous combustion oxidation reaction is not controlled by a single reaction mechanism. The whole process involves moisture and gas evaporation and desorption (I), oxidative cracking (II), intense combustion stage (III), and activation phase transition (IV). For exploring the mechanism of coal spontaneous combustion, only the second and third stages need to be considered, namely the oxidative cracking stage and the combustion process. For different reaction mechanisms, the selection of the integral function *M*(*α*) corresponding to the kinetic model function *f*(*α*) has different forms [24].

Taking the maximum constant rate point as the limit, the spontaneous combustion of the gangue is divided into two stages fitting equations: low-temperature oxidation II and high-temperature combustion III. Compare and analyze the consistency of the calculation results of the two kinetic calculation methods under the condition of nonisothermal heating rate. If the selected reaction model is reasonable, the values of *E* and *A* (or ln *A*) obtained by the two calculation methods should be similar. The values of parameters *E* and A should be in the normal value range of the kinetic parameters of energetic materials, and the coefficient *R*^2^ of the fitting degree should be above 0.98. It is considered that the oxidation process of the coal gangue conforms to the kinetic model function.

## 3. Results and Discussion

### 3.1. Coal Gangue Reaction Kinetics Solution

Activation energy refers to the lowest energy that a coal gangue can undergo oxidation reaction at. At a certain temperature, the energy of the activated molecule is higher than the average energy of other molecules, and it is these energies that exceed the average energy to hit the original molecule, weaken or destroy its internal chemical bonds, and rearrange the atoms in the molecule to form new products. If the energy of the impacting molecule is less than this energy, the reaction cannot take place. For different substances or the types of reactions, the energy levels required for their reactions are not the same as activation energy. In other words, the larger the activation energy, the smaller the tendency of the coal gangue to spontaneously ignite. The smaller the activation energy, the easier it is to spontaneously ignite.

According to the analysis results of coal gangue thermogravimetric experiments, the Achar differential method and the Coats-Redfern integral method were used to analyze the kinetics of the second stage (116.2–353.0°C) of the spontaneous combustion oxidation of the coal gangue, and the oxidation kinetic parameters of this stage under the experimental conditions were calculated. Substitute the relevant data into the commonly used solid reaction mechanism functions to obtain the activation energy and the pre-exponential factor of the differential and integral methods, respectively. The results are shown in Table 1.

**Figure 1:**
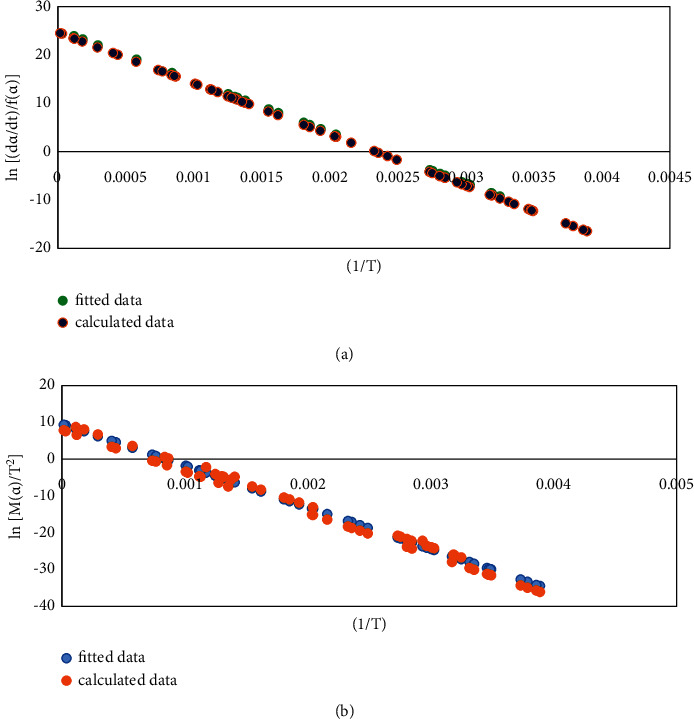
Fitted data of Avrami−Erofeev function for the second stage of coal gangue. Differential FOR (a) and integral FOR (b).

According to the inference of the Bagchi method of the reaction mechanism function, the linear correlation coefficients *R*^2^ obtained by the Avrami−Erofeev function is relatively high, which are 0.987 and 0.997, respectively, and the obtained *E* and ln*A* values are relatively close. After a comprehensive comparison, the Avrami−Erofeev function was selected for the most probable mechanism function of the second stage of the coal gangue. Substitute the experimental data of the second stage of the coal gangue into *f*(*α*) and *G*(*α*) of the Avrami−Erofeev function, plot, and fit, respectively, and for results, see Figure 1.

**Figure 2:**
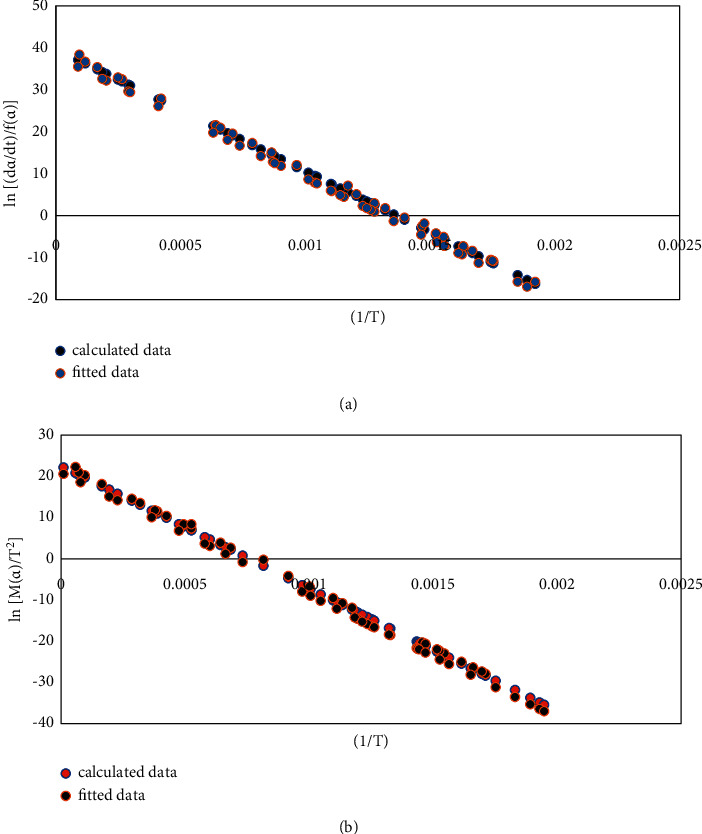
Fitted data of Avrami−Erofeev function for the third stage of coal gangue. Differential FOR (a) and integral FOR (b).

In the same way, the kinetic parameters of the third stage of the coal gangue samples were solved, and the kinetic analysis of the third stage of the coal gangue combustion was carried out. For results, see Table 2. According to the Bagchi method of the reaction mechanism function, the values of *E* and ln *A* obtained by the Avrami-Erofeev function are relatively close, and their linear correlation coefficient, R2,is 0.997 and 0.998, respectively., After comprehensive comparison, we choose the Avrami-Erofeev function as the most probable mechanism function of coal gangue stage III. Substitute the experimental data of coal gangue stage III into *f* (*α*) and *G* (*α*) of Avrami-Erofeev and plot and fit them, respectively, as shown in Figure 2.

### 3.2. Analysis of Kinetic Parameters of Coal Gangue Oxidative Combustion

According to the method and procedure of the thermodynamic analysis of 2# coal gangue sample, the Achar differential method and Coats-Redfern integral method are used to carry out the thermodynamic calculation and analysis of the coal gangue stage II and III, and the most probable reaction mechanism is deduced according to the Bagchi method. The thermodynamic parameters of 2#, 4#, 8#, and 12# coal gangue are obtained as shown in Table 3.

**Figure 3:**
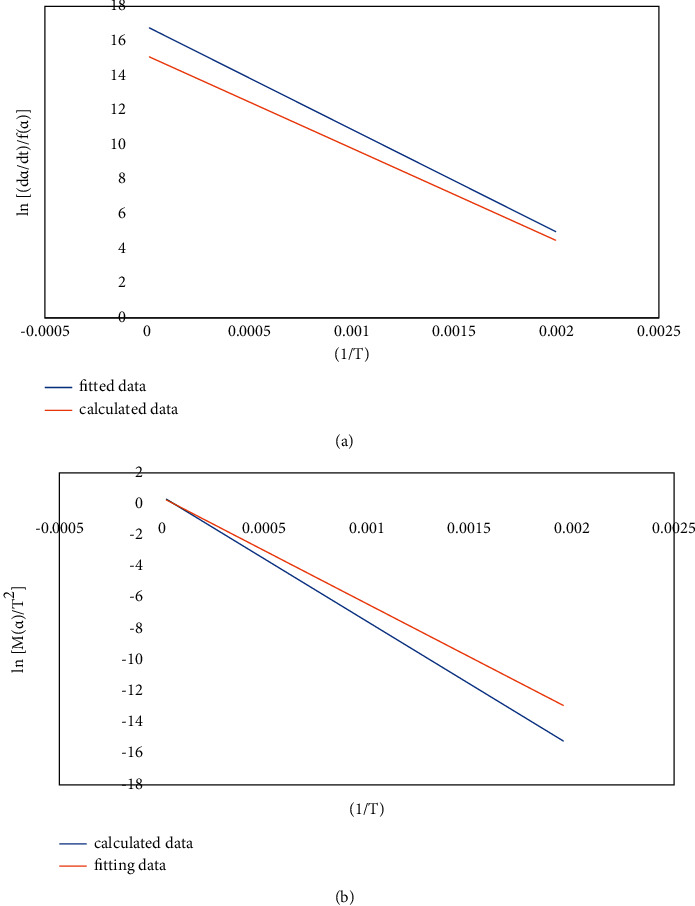
ln[(d*a*/d*t*)/*f*(*a*)], ln[*M*(*a*)/*T*^2^], and 1/*T* curves of coal gangue 4# Phase II. Reaction order function with differential form FOR (a), *R*^2^ = 0.965, with integral form FOR (b), *R*^2^ = 0.978.

**Figure 4:**
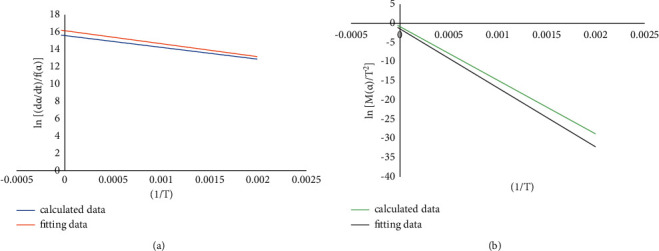
ln[(d*a*/d*t*)/*f*(*a*)], ln[*M*(*a*)/*T*^2^], and 1/*T* curves of coal gangue 4# Phase III. Avrami−Erofeev function with differential form FOR (a), *R*^2^ = 0.989, with integral form FOR (b), *R*^2^ = 0.991.

For the 4# coal gangue sample, according to the Bagchi method of the reaction mechanism function, after comprehensive comparison, the reaction order function is selected as the most probable mechanism function of the second stage of the coal gangue, and the Avrami-Erofeev function is selected as the most probable mechanism function of the third stage of the coal gangue. Substitute the experimental data of the second and third stages of the coal gangue into *f*(*α*) and *G*(*α*) of the reaction order and plot and fit them, respectively, as shown in Figures 3 and 4, respectively.

Because of the characteristics of low volatile content and high ash content of Gongwusu coal gangue, according to the kinetic parameters of the second and third stages of the coal gangue, in the initial stage of oxidative spontaneous combustion, the temperature is low, the decomposition and precipitation rate of volatile matter is low, and the ash content is high. To a certain extent, it hinders the precipitation of volatile matter in the coal gangue. Hence, the smooth oxidation reaction needs to consume a lot of energy, the activation energy and frequency factor are relatively high, the fixed carbon in the coal gangue starts to burn, the activation energy is relatively low at this time, and the oxidation reaction is easy to proceed.

### 3.3. Analysis of Comprehensive Combustion Characteristics of Spontaneous Combustion Coal Gangue

The ignition temperature of the coal gangue is much lower than the ignition temperature of coal with poor combustion performance in the mixture. The higher the volatile content in the coal gangue, the lower the characteristic temperature of pyrolysis, the faster the rate of volatile analysis, and the greater the total weight loss rate after pyrolysis. The fuel ratio of the coal gangue, i.e., the ratio of the fixed carbon to volatile matter (*FC*_ad_/*V*_ad_), is used to qualitatively judge the combustion performance of the coal gangue. The smaller the fuel ratio, the better the combustion performance of the coal gangue. NIE Qi−hong defined the comprehensive combustion characteristic index Sn to evaluate the combustion performance of coal [25]. For the slow-burning process of coal, the burning rate is expressed approximately with Arrhenius's law, and (14) is its definition.

(14) $$\begin{array}{c} \frac{dv}{d\tau }=A\;\exp\left(\frac{-E}{RT}\right), \end{array}$$

where *dv*/*dτ* is the combustion rate, %/min. *A* is the pre-exponential factor, and *E* is the activation energy, kJ/mol. *T* is the particle temperature, and *R* is atmospheric constant, 8.31 J (mol K).

Derivate (14) as follows:

(15) $$\begin{array}{c} S_{n}=\frac{{dv/d\tau }_{\max}{dv/d\tau }_{ave}}{T_{i}^{2}T_{h}}, \end{array}$$

where *dv*/*dτ*_max_ is the maximum combustion rate, %/min. *dv*/*dτ*_ave_ is the average burning rate of the combustibles, %/min. *T*_*i*_ is fire temperature, K. *T*_*h*_ is the burnout temperature. Ignition index (*D*_i_) and extinction index (*D*_*h*_) are calculated by equations (16) and (17), respectively. They can evaluate the ignition and extinguishing characteristics of the gangue.

(16) $$\begin{array}{c} D_{i}=\frac{DT\;G_{\max}}{t_{p}t_{i}}. \end{array}$$

(17) $$\begin{array}{c} D_{h}=\frac{DT\;G_{\max}}{\Delta t_{1/2}t_{p}t_{h}}, \end{array}$$

where *DT*  *G*_max_ is the maximum burning rate, *t*_*p*_ is the corresponding time at *DT*  *G*_max_, ignition time of *t*_1_=*t*_3_, Δ*t*_1/2_ is the time when *DT*  *G*/*DT*  *G*_max_=0.5, *t*_*h*_=*t*_5_, *T*_*i*_=*T*_3_, °C, and *T*_4_ is the maximum combustion rate of the combustible substances that corresponds to the temperature. Combined with the results of the thermogravimetric test of the coal gangue and coal, according to formulas 15−17, the characteristic parameters of the oxidative combustion process of the coal gangue and coal are calculated as shown in Table 4.

We can conclude from Table 4 that the comprehensive combustion index *S*_4_ > *S*_8_ > *S*_12_ > *S*_2_ > *S*_13_. Among the 5 coal gangues, 4# coal gangue has the largest comprehensive combustion index, high volatile content, low pyrolysis characteristic temperature, faster volatile analysis rate, and the largest total weight loss rate after pyrolysis. It is corroborated by the experimental results. The corresponding 2# coal gangue has high lime content, low volatile and fixed carbon content, and its comprehensive combustion index is the smallest, which is not easy to burn. (2) Ignition index *D*_*i* 8_ > *D*_*i* 12_ > *D*_*i* 4_ > *D*_*i* 2_. Among the 5 coal gangues, the ignition index of 8# is the largest, which is better than that of 12#, 4#, and 2# coal gangue. Industrial analysis shows that the 8# coal gangue has a high fixed carbon content, and its ignition index may be related to its fixed carbon content. (3) Burnout index *D*_*h* 12_ > *D*_*h* 8_ > *D*_*h* 2_ > *D*_*h* 4_. The larger the burnout index, the worse the burnout performance. Among the 5 coal gangue samples, 12# has the worst burnout performance, 8# is the second, and 4# has the best burnout performance. It shows that in the later stage of coal gangue combustion, the ash content hinders the diffusion of oxygen molecules in the direction of combustible substances to a certain extent. Therefore, the higher the content of fixed carbon in the coal gangue, the worse the burnout performance.

The characteristic parameters and comprehensive combustion characteristics of coal gangue in different regions and ages are compared, as shown in Table 5.

We can demonstrate from Table 5 that the ignition temperature *T*_5_ of Gongwusu coal gangue used in the test is 410.2°C, the lowest ignition temperature, and the highest ignition temperature of Hancheng coal gangue indicating that the coal gangue contained in Gongwusu coal gangue has a low ignition point, and the coal gangue is prone to ignition. Under the condition that the particle size of the coal gangue is not much different, comparing the comprehensive combustion index S of the coal gangue in different regions, it can be seen that the comprehensive combustion index of the Gongwusu coal gangue used in the test is higher, which is 31.02 × 10−31.02∗11%min-2K-3. It is second only to the coal gangue in the Datong area and higher than the coal gangue in the Panzhihua, Pingdingshan, and Hancheng areas, indicating that the Gongwusu coal gangue has better characteristics of burning and burning out. According to the industrial analysis data of the coal gangue, it is found that the level of volatile matter content and the comprehensive combustion characteristic index *S* are consistent. The ratio of fixed carbon to volatile matter in the coal gangue used in the test is 0.93, which is the lowest among the five coal gangues, and its weight loss rate of 32.5% is the highest among the five coal gangues, which shows good performance of burnout and the high total weight loss rate of oxidative combustion.

### 3.4. Study on Heat Transfer Method of Hot Rod in Gangue Mountain

#### 3.4.1. Thermal Behavior Characteristics of Coal Pile Temperature Field under the Influence of a Single Hot Rod

Coal gangue mountain spontaneous combustion is a relatively special combustion system, and its ignition, combustion maintenance, fire zone transfer, and combustion extinguishing are very different from ordinary fires. According to the unique heat conduction characteristics of the hot rod to eliminate the heat storage environment of the coal gangue mountain, it can destroy the spontaneous combustion conditions and prevent the spontaneous combustion from occurring.

Where there is a temperature difference, there must be a natural phenomenon of heat transfer from high temperature to low temperature. Of the three ways of heat transfer—heat radiation, convection, conduction,and the rate of heat transfer is the fastest. The hot rod is a heat exchange element with extremely high heat transfer efficiency. It relies on the internal working fluid phase change and continuous working fluid convective heat transfer and circulation to achieve efficient heat transfer. It is the device with the highest heat transfer efficiency in passive cooling systems. Hot rods are also called heat pipes because they are generally larger in mining engineering. Hence, they are called hot rods or hot piles.

In our study, we tested the influence of a single hot rod on the thermal behavior of the coal pile, and the temporal and spatial variation of the coal pile temperature field with and without the hot rod was compared and analyzed. The hot rod is 100 mm away from the heating surface in the *X* direction. The initial temperature of the experiment is set to 100°C, and the data is read and observed every 10 hours. We conducted the temperature response inside the coal pile, and its influence on the temperature field were tested under the action of two hot rods. Observations determine the behavioral effect of the distance between the two hot rods on the temperature field of the coal pile. The buried depth of the evaporation section of the hot rod is 490 mm. The distance between the hot rod and the heating surface in the *X* direction is 100 mm, and the distance between the two hot rods is 100 mm, 200 mm, and 300 mm. We read and observe data every 10 hours. Finally, the enhanced cooling effect of the hot rod on the coal pile was studied under different heating temperature conditions. The buried depth of the evaporation section of the hot rod is 490 mm, and the distance between the hot rod and the heating surface in the *X* direction is 100 mm. Three groups of initial heating temperatures of 100, 150, and 200°C are selected for testing, and the data is read and observed every 10 hours. The data processing method of this experiment is mainly to compare the slope of each group of curves (the rate of temperature change) and the cooling range. This method can intuitively see the size of the heat exchange and the heat exchange efficiency of the heat pipe under different conditions.

**Figure 5:**
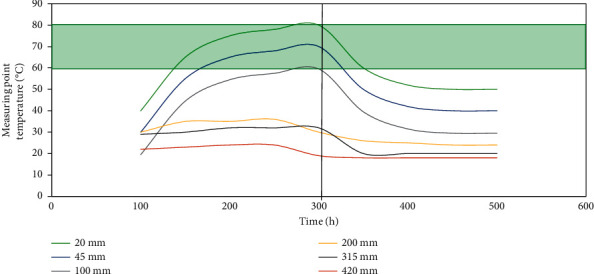
Variation of coal pile temperature curve with time. The shaded part represents the critical temperature range of coal spontaneous combustion. 0∼300 h is without the hot rod stage, and 300∼600 h is with the hot rod stage.

To study the effect of hot rods on the distribution of the temperature field inside the coal pile, the changes in the temperature field inside the coal pile before and after the effect of the hot rods were observed experimentally under the condition that there is an ignition source inside the coal pile, as shown in Figure 5.

The conclusion from Figure 5 shows that the flat plate heater is set to a constant temperature of 150°C to simulate the temperature of the fire source to heat the coal pile, and the temperature of the coal pile gradually rises without the action of the hot rod. The faster the rate, the higher the temperature at No. 1 measuring point, reaching about 84°C. After heating for 300 hours, the temperature of each measuring point in the coal pile is stable. At this time, the heat input of the heater to the test coal pile and the heat dissipation of the coal pile to the environment are in a dynamic balance, and the temperature field inside the coal pile is in a relatively stable state. After the temperature of the coal pile is stable, insert the hot rod into the predetermined position of the coal pile. It can be seen from Figure 5 that the monitoring temperature in the coal pile drops rapidly after the hot rod is inserted. After inserting the hot rod for about 50 h (the test time is about 350 h), the temperature of the coal pile gradually stabilized again. Among them, the temperature drop of No. 1, 2, 3, and 4 points was obvious because of the enhanced heat dissipation effect of the hot rod, and the temperature of No. 5 test point decreased significantly. The temperature change at the No. 6 measuring point is not obvious. It is generally believed that the critical temperature range for the coal spontaneous combustion is 60–80°C. When the coal temperature is lower than the critical temperature of spontaneous combustion, the oxygen consumption rate and oxidation heating rate of coal increase slowly. When the temperature of coal is higher than the critical temperature of spontaneous combustion, it is regarded as the spontaneous combustion of coal.

**Figure 6:**
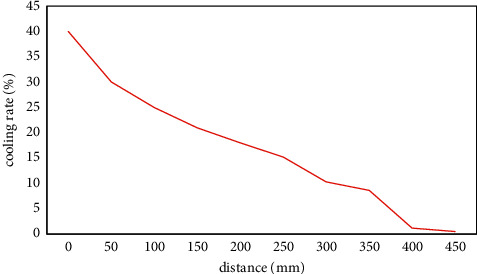
The cooling effect of each measuring point under the action of the hot rod.

After the test was carried out for 460 hours (the working time of the hot rod was about 150 h), the temperature in the coal pile became stable again, and the temperature data was processed to analyze the enhanced cooling effect of the hot rod on the coal pile under the test conditions.  Figure 6 shows the cooling range and cooling rate of each measuring point in the coal pile under the action of the hot rod. It can be seen that the hot rod has the most obvious effect on the temperature of the No. 1 measuring point, where the cooling range of the coal pile is 33.4°C, and the cooling rate reaches 39.6%. The cooling effect here is weak. The results show that the cooling effect of the hot rod on the coal pile is inversely proportional to the distance between the hot rod and the coal body. The closer the hot rod to the coal body, the greater the cooling range of the coal body. As the distance from the edge of the hot rod increases, the influence of the hot rod on the distribution of the temperature field inside the coal pile gradually decreases.

#### 3.4.2. Thermal Behavior Characteristics of Coal Pile Temperature Field under the Influence of Double Hot Rods

**Figure 7:**
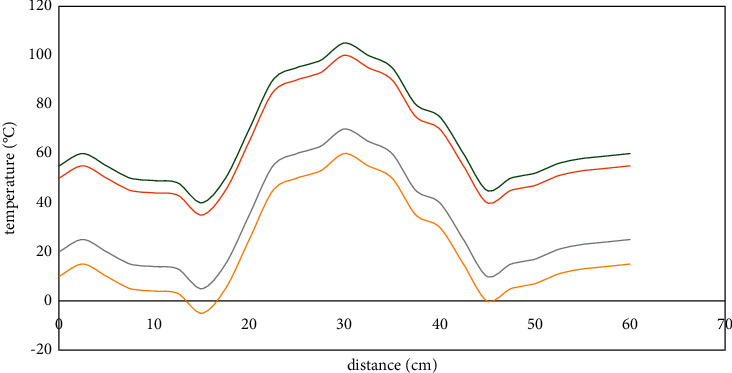
.Temperature changes of two hot rods at different times.

Figure 7 is the temperature change curve of the coal pile with two hot rods arranged at 10 cm parallel to the heating plate. With the increase in heating time, the overall temperature of the coal pile is on the rise, and the temperature rise rate is the fastest in the first 10 hours and the amplitude is the largest. After that, the heating rate gradually decreases with the increase of time, which is similar to that of the coal pile under the action of a single hot rod. The law of temperature field changes is consistent. In addition, the curve in the figure presents a “saddle” shape, because at the center point (*x* = 30 cm) of the line connecting the two hot rods, the temperature of the coal pile is up to 105°C. However, under the action of the external heat source for 132 hours at the central part of the installation of the hot rod (see Figure 7), the temperature of the coal body is still lower than 50°C. The cooling effect of the hot rod weakens with the increase of the radius, and the coal pile at the center point of the connection line is difficult to reduce rapidly in a short time. Therefore, the temperature field in the coal pile has a “saddle” shape because of the superposition of heating.

**Figure 8:**
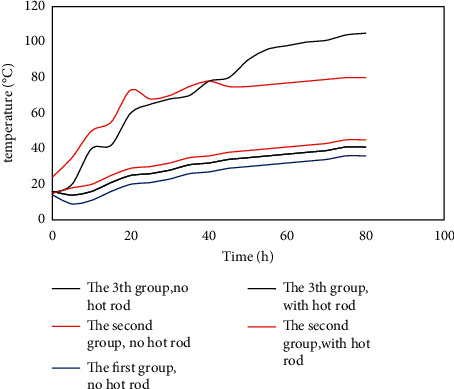
Temperature curve of No. 1 measuring point with time under different heat source temperature conditions. The bold line is the experimental group without a hot rod, and the thick line is the control group with a hot rod.

Once the coal temperature exceeds 200°C, the oxidation reaction rate of the coal is accelerated, and the spontaneous combustion stage quickly enters the combustion stage. To test the effect of the hot rod on the internal temperature field of the coal pile under different fire source temperature conditions, the initial temperature of the plate heater was set to 100, 150, and 200°C to simulate the heat source temperature. The internal temperature of the coal pile is changed with time when the coal pile is heated for 80 hours with hot rods (control group) and without hot rods (normal group). Based on the analysis of the heat transfer performance of the hot rod, it can be seen that the closer the coal pile is to the hot rod, the more obvious the effect of the hot rod on the temperature of the coal pile. Hence, the No. 1 measuring point is selected for analysis. The variation curves of coal pile temperature with time obtained from the three groups of tests are shown in Figure 8.

It can be seen from Figure 8 that under the different conditions of the initial heating temperature of the three groups, with the increase of time, the temperature of the coal pile is on the rise as a whole. During the heating process within 80 hours, the temperature of the measuring point in the normal group (without the hot rod) rose to a maximum of around 46°C, and the temperature of the *b* normal group and *c* normal group reached around 78°C and 104°C, respectively. The reason for the analysis is that the temperature of the heat source in the normal group *a* is low, and the temperature at the measuring point after heating is still lower than the critical temperature, while the normal groups *b* and *c* are heated without a hot rod. The temperature of the measuring point rises rapidly and exceeds the critical temperature. It can be considered that spontaneous combustion of coal has occurred there. Under the action of the hot rod, the temperature of the coal pile was kept in a low range in the three groups of tests, and the maximum coal temperature did not exceed 60°C. It shows that the hot rod can control the temperature of the coal body below the critical temperature, which can remove the heat inside the coal pile, delay the spontaneous combustion process of the coal, and prevent the spontaneous combustion of the coal body.

**Figure 9:**
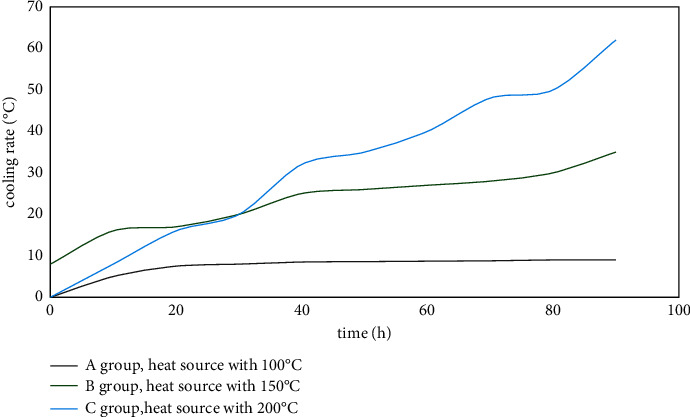
Variation of the cooling amplitude value of no. 1 measuring point with time at different heating temperatures.

Figure 9 is a graph of the cooling range of the coal pile under different heating temperatures. The greater the intensity of the heat source, the greater the cooling rate of the hot rod to the coal pile. In group *a* (heat source temperature of 110°C), the cooling range of the coal pile was maintained at 6−7°C. In group *b* (heat source temperature of 150°C), the cooling range was maintained at 25−28°C. In the heat source temperature of 200°C, the cooling amplitude value increases the fastest, and the cooling amplitude can reach up to 57.5°C. With the increase of time, the cooling amplitude has a further increasing trend. Analyzing the reasons, the heating temperature of group *a* is low, the heat flow inside the coal pile is small, and the heat dissipation power of the hot rod is also small. When the temperature is 200°C, the power of the hot rod is the largest among the three groups of tests, and its heat dissipation capacity to the coal pile is also the strongest. It shows that the cooling ability of the hot rod to the coal pile increases with the increase of the internal temperature of the spontaneous combustion coal pile.

As an index to quantitatively evaluate the cooling effect of the hot rod on the coal pile, the heat dissipation is of great significance. The amount of heat dissipation is related to the structure of the hot rod, and the cooling effect of the hot rod on the coal pile is also related to the particle size, porosity, thermophysical properties, air temperature, and internal temperature of the coal pile.

**Figure 10:**
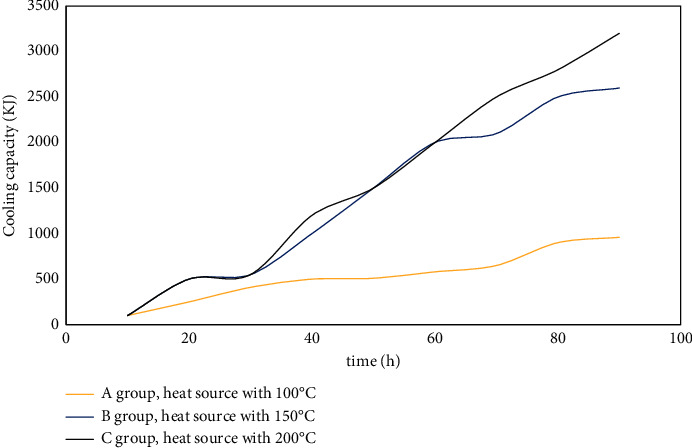
Variation curve of heat dissipation of hot rod with time under different heat source temperature conditions.

The change of heat dissipation trend can objectively evaluate the performance of the hot rod [26]. The heat dissipation of the hot rod is proportional to the temperature difference between the coal pile and the environment and the working time of the hot rod, and it is inversely proportional to the thermal resistance of the hot rod. During the test, the average indoor temperature was 11°C, and the test time was 80 h. The average temperatures of the coal piles under the three groups of test conditions were 39.82, 42.11, and 52.18°C, respectively, and the total thermal resistance of the coal pile-hot rod system was 5.0263°C/W. According to the formula (15), the cooling capacity of the hot rod under the test conditions is obtained as shown in Figure 10.

## 4. Conclusions

In our study, we first solved the dynamic parameters and mechanical functions of the coal gangue using the Achar differential method and Coats-Redfern integral method, analyzed the comprehensive combustion characteristics of the coal gangue, and then, based on the flow and heat transfer mechanism of hot rod vapor-liquid two-phase flow, combined with coal spontaneous ignition conditions, influencing factors, coal pile spontaneous combustion temperature field structure distribution, etc., the heat transfer process of hot rod in coal pile (coal gangue mountain) was analyzed. The excellent characteristics and working characteristics of the hot rod are studied, the thermal turnover process of the hot rod in the coal pile is studied, the thermal resistance network of the hot rod and the mathematical expression of the heat transfer power are analyzed, and the unsteady mathematical model of the heat transfer of the hot rod is constructed. The quantitative standard for evaluating and judging the thermal turnover effect of the hot rod is studied. The main conclusions are as follows.

1. The average activation energies of the second stage of 2#, 4#, 8#, and 12# coal gangue are 88.664, 48.365, 105.345, and 189.63 kJ/mol, respectively. According to the Bagchi method, the most probable mechanism functions of the second stage are inferred as Avrami−Erofeev equation, reaction order equation, and Avrami−Erofeev equation with different stages; the average activation energies of the third stage are 253.557, 115.526, 354.594, and 398.454 kJ/mol, respectively, and all of the machine functions are Avrami−Erofeev.
2. Upon comparing the characteristic parameters of the coal gangue in different regions and ages, it is found that 8# Gongwusu coal gangue has better combustion and burn-out characteristics, and its comprehensive combustion characteristics are second only to those of the coal gangue in Datong, and they are higher than those in Panzhihua, Pingdingshan, and Hancheng. The ratio of the fixed carbon to the volatile matter of 8# Gongwusu coal gangue is 0.88, which is the lowest among the five different coal gangues compared. Its weight loss rate is 30.30%, which is the highest among the five coal gangues, indicating that the volatile analysis of 8# coal gangue has fast speed, easy to ignite and burn, good burnout performance, the high total weight loss rate of oxidative combustion, and low ignition point of coal contained, which is prone to spontaneous combustion.
3. The correlation between *S*_*n*_ and (*V*_*a*  *d*_ + *FC*_*a*  *d*_)/*A*_*a*  *d*_ is positive. It represents that the higher the content of volatile matter and fixed carbon in coal gangue, the lower the ash content, and the better the comprehensive combustion performance of the coal gangue. The comprehensive combustion index, ignition index, and burnout index were positively correlated with *FC*_ad_/*V*_ad_. The burnout index and the linear correlation of the fuel ratio *FC*_ad_/V_*ad*_ is the highest, followed by the ignition index, and the comprehensive combustion index is the lowest. Under the condition of sufficient oxygen supply combustion, the larger the fuel ratio, the better the burnout performance of the coal gangue.
4. The test of the influence of the hot rod on the temperature field distribution inside the coal pile shows that the maximum cooling rate of a single hot rod to the coal pile during the test period is 33.4°C, and the maximum cooling rate reaches 39.6%. However, under the action of the external heat source for 132 hours, the temperature of the coal body was still lower than 50°C. In the three sets of tests with different heating powers, as the temperature of the heat source increases, the heat flux density in the coal pile increases, and the heat dissipation power of the hot rod also increases.
5. The calculated heat dissipation of the 80 h hot rod is 1.0865, 2.1680, and 3.3649 MJ, respectively. The increase in the heat dissipation of the hot rod can strengthen the cooling efficiency of the coal pile, effectively reduce the internal temperature of the coal pile, and thus inhibit the spontaneous combustion of the coal pile. The cooling effect of the hot rod on the coal pile is inversely proportional to the distance between the hot rod and the coal body. The closer the hot rod is to the coal body, the greater the cooling range of the coal body. The cooling ability of the hot rod to the coal pile increases with the increase of the internal temperature of the spontaneous combustion coal pile. The heat dissipation of the hot rod has been increasing with the increase of time, indicating that the cooling capacity of the hot rod to the coal pile will continue to increase with time.

## Figures and Tables

**Table 1:** Phase II kinetic parameters of coal gangue at a heating rate of 20°C/min.

Function form	Differential			Integral		
	*E*	ln*A*	Correlation *R*	*E*	ln*A*	Correlation *R*
Avrami−erofeev equation	89.996	25.321	0.987	96.211	20.997	0.997

**Table 2:** Phase III kinetic parameters of coal gangue at a heating rate of 20°C/min.

Function form	Differential			Integral		
	*E*	ln*A*	Correlation *R*	*E*	ln*A*	Correlation *R*
Avrami- erofeev function	250.996	36.896	0.997	261.125	36.559	0.998

**Table 3:** Kinetic parameters of the second and third stages of coal gangue at a heating rate of 20°C/min.

Sample	Oxidation stage	Mechanism function	Activation energy (kJ·mol^−1^)	Pre-exponential factor (log *A*/s^−1^)	Relationship
2	II	Avrami−Erofeev	88.664	28.662	0.993
	III	Avrami−Erofeev	253.557	45.325	0.994

4	II	Reaction order	48.365	20.162	0.991
	III	Avrami−Erofeev	115.526	19.632	0.986

8	II	Avrami−Erofeev	105.345	36.23	0.988
	III	Avrami−Erofeev	354.594	66.335	0.998

12	II	Avrami−Erofeev	189.63	55.632	0.996
	III	Avrami−Erofeev	398.454	67.562	0.995

**Table 4:** The results of the characteristic parameters of the oxidative combustion process of coal gangue and coal.

Number	*dv*/*dτ*_max_ (%min^−1^)	*dv*/*dτ*_ave_(%)	*D*_*i*_ × 10^4^	*D*_*h*_ × 10^4^	*S*_*n*_(10^11^%^2^)
2	−0.862	−0.421	14.325	0.896	4.985
4	−1.455	−1.201	17.987	0.621	29.221
8	−2.181	−0.721	40.220	2.067	29.125
12	−1.775	−0.521	32.667	2.981	16.541
13	−6.895	−1.584	200.215	6.785	211.952

**Table 5:** Characteristic parameters and combustion index of coal gangue in different regions.

Types of coal gangue	Total weight loss (%)	*T*_5_(%°C)	*V*_*a* *d*_(%)	*FC*_*a* *d*_/*V*_*a* *d*_	*S*_*n*_ × 10^11^(%^2^/min · K^3^)
Experimental coal gangue 8#	32.5	410.2	16.35	0.93	31.02
Pingdingshan coal gangue	16	420	9.1	1.3	9.1
Datong coal gangue	25	400	18.2	1.1	39.2
Hancheng coal gangue	23	460	6.88	4.1	9.1
Panzhihua coal gangue	22	450	10.26	1.1	13.5

## Data Availability

The experimental data used to support the findings of this study are available from the corresponding author upon request.
